# Changes in diffusion MRI and clinical motor function after physical/occupational therapies in toddler-aged children with spastic unilateral cerebral palsy

**DOI:** 10.3389/fneur.2024.1418054

**Published:** 2024-10-09

**Authors:** Adam Bernstein, Heidi Pottinger, Jeffrey Miller, Unni Udayasankar, Theodore Trouard, Burris Duncan

**Affiliations:** ^1^Department of Biomedical Engineering, University of Arizona, Tucson, AZ, United States; ^2^Department of Health Promotion Sciences, MEZ College of Public Health, University of Arizona, Tucson, AZ, United States; ^3^Office of Community Health, Engagement, & Resiliency, Arizona State University, Tucson, AZ, United States; ^4^Department of Radiology, Phoenix Children’s Hospital, Phoenix, AZ, United States; ^5^Department of Medical Imaging, University of Arizona, Tucson, AZ, United States; ^6^Sonoran Center of Excellence in Disabilities, University of Arizona, Tucson, AZ, United States

**Keywords:** diffusion, magnetic resonance imaging, cerebral palsy, physical therapy, occupational therapy

## Abstract

Diffusion-weighted magnetic resonance imaging (DMRI) is a potential tool to assess changes in brain connectivity and microstructure resulting from physical and occupational therapy in young children with cerebral palsy. This works was carried out to assess whether DMRI can detect changes after 36 weeks of physical and occupational therapy in the microstructure and connectivity of the brains of children with cerebral palsy and determine whether imaging findings correlate with changes in clinical measures of motor function. Five children underwent anatomical MRI and DMRI and evaluations of motor function skills at baseline and after 36 weeks of intensive or once-weekly physical and occupational Perception-Action Approach therapies. Diffusion tensor imaging and constrained spherical deconvolution methods were used to calculate fractional anisotropy (FA) and fiber orientation distribution functions (fODFs), respectively. The fODFs were used to generate tractograms of the cerebrospinal tract (CST). After 36 weeks of physical and occupational therapy, all children showed increases in motor function. No changes were observed in anatomical MRI before and after therapy but CST tractography did show small differences indicating possible altered microstructure and connectivity in the brain. FA values along the CSTs, however, showed no significant changes. Reliable longitudinal DMRI can be employed in toddler-aged children with CP and DMRI has the potential to monitor neuroplastic changes in white matter microstructure. However, there is a high variability between subjects and clinical improvements were not always correlated with measures of FA along the CST.

## Introduction

Cerebral Palsy (CP) is a descriptive term for a group of disorders of movement and posture causing limitations or dysfunction of activity. The etiology is a non-progressive insult or injury to the central nervous system (CNS) in the fetal or neonatal brain (1). The motor defect is often progressive. CP is the most frequent cause of childhood disability with overall prevalence rates in the U.S. of 3.3 per 1,000 live births (2). Whereas there is no cure for CP, there are multiple treatment options that, depending on severity can improve the child’s function and quality of life (3). Most treatments involve different types of physical therapy that addresses the primary peripheral motor deficit (4). The children presented in this case series were part of a larger clinical trial that was designed to evaluate the effectiveness of an intensive regimen of physical (PT) and occupational therapies (OT), using the Perception–Action Approach (P-AA) (5), in children with mild-to-moderate spastic CP between 12 and 36 months of age. P-AA is an active (not passive), child-driven therapy with the therapist enhancing environmental stimuli to assist and improve the child’s active movement. P-AA utilizes current principles of neuroplasticity and adaptability of movement. The reorganizing of neural tissue has the greatest potential when the brain is young and when the activity is active, frequent, and repetitive. Reorganization can be done in several ways, including the recruitment of undamaged neurons adjacent to injured neurons, recruitment of neurons from supplemental non-primary motor areas, and stimulation of ipsilateral neurons (6).

Magnetic resonance imaging (MRI) is a particularly important tool for assessing the structure of the brain, particularly in children, because it is non-invasive and uses no ionizing radiation (7–11). In CP, MRI is typically used to identify the extent and location of damage and to identify specific pathologies, such as brain malformations, hemorrhage, and periventricular leukomalacia. Diffusion MRI (DMRI), and particularly diffusion tensor imaging (DTI), is a promising tool for characterizing changes in white matter microstructure in CP in both cross-sectional studies (12, 13) and longitudinal studies assessing the efficacy of different therapies (14). Additionally, DMRI allows for the assessment of structural connectivity, which has also been used for characterizing organizational changes in the brain in individuals with CP (12, 13).

In this brief research report, we present the results of DMRI carried out in five children with spastic unilateral CP before and after therapy. The therapy employed P-AA in an outpatient setting from trained PT and OT therapists and by caregivers who were instructed by therapists in administering P-AA task-oriented activities at home. Both DTI (15) and constrained spherical deconvolution (CSD) (16) parameters were assessed before and after 36 weeks of therapy to evaluate changes in brain microstructure. Correlations between DMRI metrics and clinical outcomes were assessed, as well as organizational changes in the brain.

## Materials and methods

### Subjects

The five subjects in this small imaging study were a convenience sample of children who had hemiparesis and were recruited from a larger clinical trial designed to investigate the effects of intensive PT and OT in toddler-aged children with mild–moderate spastic CP (17). Participants were recruited from trial sites at Tucson Medical Center and Phoenix Children’s Hospital (PCH). All imagining studies were carried out at PCH. Inclusion criteria for the clinical trial included ages between 12 and 36 months at enrollment, a diagnosis of spastic CP confirmed by a pediatric neurologist or pediatric rehabilitation specialist, and a Gross Motor Function Classification System (GMFCS) severity level of I, II, or III (18). The central nervous system insult causing CP must have occurred either during gestation or within 1 year after birth, independent of gestational age. Subjects were excluded based on: a diagnosis of CP secondary to neuronal migration; the presence of any medical condition preventing the administration of rehabilitation therapies or the administration of the evaluation tool at the intensity required by the study; and involvement in any co-interventions, such as a pharmacological intervention or procedure (17). This small sub-study was approved by the PCH and the University of Arizona (overseeing Tucson Medical Center) Institutional Review Boards and written informed consent was obtained from a parent for all subjects.

### Physical therapy

The children received a varying number of outpatient PT and OT sessions (1–5 sessions/week for each) over 36 weeks using the P-AA (5) plus widely varying amounts of additional P-AA simulated therapies by the parent at home. See Supplementary Table S1.

### Clinical evaluation

The clinical evaluation instruments included a physical therapy evaluation administered by a certified physical therapist familiar with the child, using the Gross Motor Function Measure 66-item test (GMFM-66) (19). The GMFM-66 evaluation was recorded on video and scored by another physical therapist, blinded to the intervention group with a post reliability of 0.99 (using a weighted kappa; passing score set at 0.80). A parental assessment of the child’s functional abilities was assessed using the Pediatric Evaluation Disability Inventory–Functional Skills scaled scores (PEDI-FS) (20). The GMFM-66 and PEDI-FS were administered the same day at baseline and 36 weeks.

The GMFM-66 is a criterion-referenced validated instrument that has been shown to detect changes in the gross motor function of children with CP following a variety of interventions. The 66 test items are grouped into five dimensions: Lying and Rolling, Crawling and Kneeling; Sitting, Standing, and Walking, Running and Jumping (19). The item set method was used and total scores were calculated using the second edition of the Gross Motor Ability Estimator (GMAE) software (21).

The PEDI is an instrument developed specifically for children from 6 months through 7 years of age who have some type of disability. It is a child’s primary care provider’s assessment of what skills the child can perform. The functional skills subset is grouped into three domains: (1) self-care, (2) mobility, and (3) social function. Total scores for each domain were converted to scaled scores provided in the manual appendices. Only mobility scores are reported here (20).

The number of therapy hours the children received in the clinic by certified therapists ranged from 31 to 81 and the number of hours the therapy was administered by the caregivers (‘co-therapists’) varied from zero-383 (see Supplementary Table S1 for details).

### Imaging data acquisition

All of the imaging was performed on a single 3 T Ingenia MRI system (Philips Healthcare, Best, the Netherlands) equipped with a 32-channel head coil. Imaging included both conventional MRI and DTI sequences. Children were anesthetized and monitored by standard clinical practice at PCH. Imaging was performed once at baseline, before therapy, and once at 36 weeks following therapy. A 3D T_1_-weighted dataset (TR = 8.9 ms, TE = 4.1 ms, flip angle = 8°, resolution = 0.9 mm x 0.9 mm x 0.9 mm, FOV = 210×210 mm^2^) was acquired for anatomical reference. T2-FLAIR images were acquired (TR = 4,800 ms, TE = 400 ms, TI = 1,650 ms, in plane matrix 480 × 480, in-plane FOV = 21 cm x 21 cm, slice thickness = 3 mm) acquired to assess white matter hyperintensities. The DMRI protocol included a 64-direction, b = 1,000 s/mm^2^ single shot EPI diffusion MRI sequence with 6 b = 0 s/mm^2^ volumes collected. The resolution of the DMRI is 2.25 mm isotropic, with a TR/TE of 8150/109 ms.

### Image preprocessing

DMRI images were preprocessed with the following steps. Gibbs ringing correction was first performed on the original DMRI images using the method described by Kellner et al. (22). Eddy current and motion correction was then performed using *FSL*’s eddy algorithm (23), and the diffusion encoding directions were adjusted for head motion (24). Local principal component analysis (LPCA) was then used to denoise the dataset (25), after which N4 bias field correction, as implemented as a part of the *ANTs* software package, was performed as previously described by Tustison et al. (26). Finally, the images of the subjects with the lesion on the right were left–right flipped such that all subjects’ lesions were on the left side of the image for ease of comparison.

### Microstructure processing

After preprocessing, all data were fit to the diffusion tensor imaging (DTI) model using a weighted linear least squares fit (15) using in house *Python* code. From the voxel-wise fitted tensors, scalar maps of fractional anisotropy (FA), and radial diffusivity (RD) were calculated from the eigenvalues of the diffusion tensors. Constrained spherical deconvolution (CSD) was also performed to estimate fiber orientation distribution functions (fODFs) on a voxel by voxel basis (16). The fODF maps generated from both imaging sessions were registered to one another for each subject in order to create an image at a midway point, such that tractography and microstructure analysis were performed in a common space per subject.

Deterministic CSD-based tractography (27) was performed to identify the corticospinal tract (CST) by seeding tracks in the cerebral peduncles and requiring all generated tracks to pass through a region of interest (ROI) drawn on the posterior limb of the internal capsule. Wakana et al. (28) suggested using an ROI drawn on the precentral gyrus (primary motor cortex), but due to large anatomical abnormalities superior to the internal capsule in two subjects, the internal capsule provided a more consistent ROI across subjects. All ROIs were drawn on each subjects’ individual template image. While tractography was performed in a common space, the registered fODFs from timepoint one and timepoint two were used to generate their respective tractograms. FA maps calculated at baseline and follow-up were transformed into each subject’s template space using the warps generated from the fODF registration, and were sampled along the length of each CST generated. This data was used to generate an along-track measure of FA.

## Results

All subjects showed an increase in GMFM-66 from baseline to week 36 varying from 6.5 to 15.8 (See Supplementary Table S2). A similar trend was seen in the PEDI mobility scores, with percent changes varying from an increase of 2 to 53% from baseline (See Supplementary Table S2).

**Figure 1:**
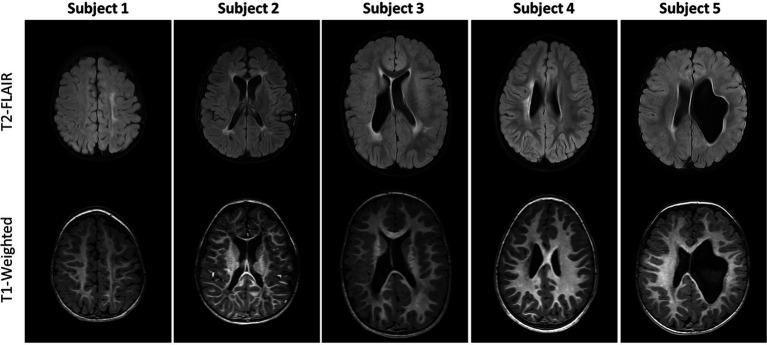
Axial T2-FLAIR (top row) and Axial unenhanced T1-weighted images (bottom row) for the five subjects studied. Different axial sections are shown so that each subject’s injuries are visible. MRI findings from these two sequences are as follows. Subject 1: Mild left white matter injury of centrum semiovale (CS), minimal left perirolandic volume loss and cortical/subcortical gliosis. Subject 2: Moderate bilateral periventricular leukomalacia (PVL) and white matter injury of the centrum semiovale, moderate volume loss and perirolandic cortical/subcortical gliosis; Subject 3: Moderate right, mild left PVL and white matter injury of the centrum semiovale, mild right volume loss; Subject 4: Moderate white matter injury of right centrum semiovale, mild volume loss and perirolandic cortical/subcortical gliosis; Subject 5: Moderate/Severe left volume loss, minimal bilateral PVL.

**Figure 2:**
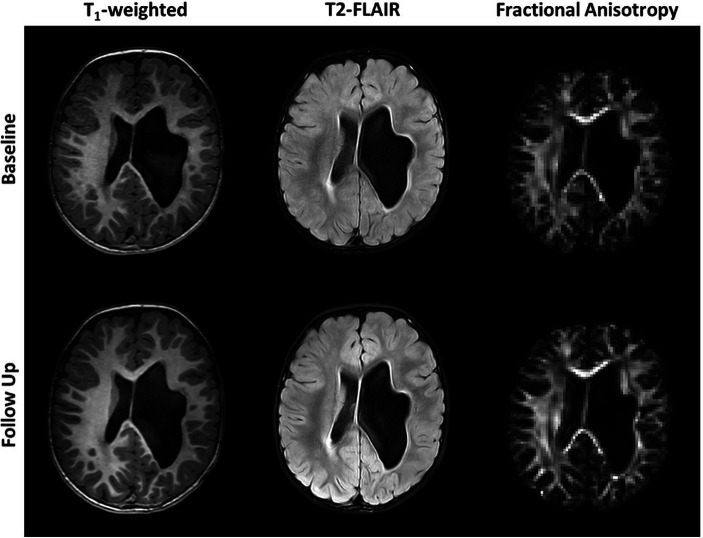
Axial unenhanced T1-weighted, T2-FLAIR images and fractional anisotropy (FA) maps of subject 5 before (Baseline) and after 36 weeks of P-AA physical therapy. No significant changes are visible in the images or maps. Subject 5 was chosen as an example and all other subjects showed identical results, where no significant changes are noticeable in the images and FA maps.

Axial T1-weighted images and T2-FLAIR images are shown in Figure 1 for all five subjects. The radiological diagnosis for each child can be found in the figure legend. As is evident in the images, the extent of injury is highly variable, ranging from mild white matter injury and minimal volume loss (subject 1) to severe white matter injury and significant volume loss (subject 5). For all subjects, there was no visible change on T1-weighted and T2-FLAIR images noted between baseline and follow-up time points. Figure 2 shows example axial MRI images from one subject (subject 5) at baseline and follow-up. Radiologically, the images at both time points show no difference. The T1-weighted images used for anatomical reference clearly delineate white matter, gray matter, and CSF, and the white matter on the affected side of the brain is hypointense relative to the white matter of the unaffected side at both baseline and follow-up. This is also true of the FLAIR images which both clearly demonstrate periventricular hyperintensity associated with periventricular leukomalacia. Finally, the FA maps derived from DTI show distinct white matter tracts, but with less volume and lower values on the affected side compared to the unaffected side. No visible changes in the FA maps were observed after therapy for any of the children.

**Figure 3:**
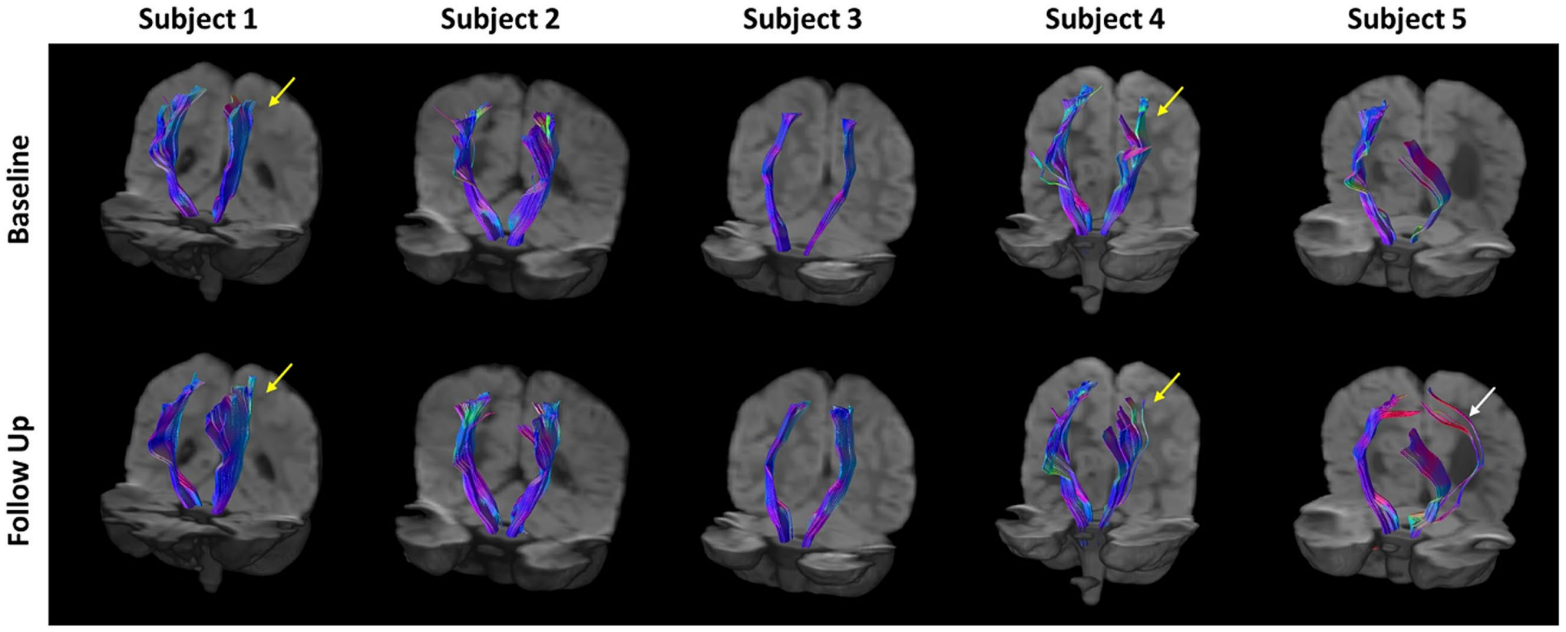
Corticospinal tract (CST) tractograms for all subject generated via deterministic tractography using fiber orientation distribution functions (fODFs) generated from constrained spherical deconvolution (CSD). There is a general increase in the breadth of the CST tractograms at follow-up compared to baseline for all subjects. Yellow arrows indicate regions of substantial reorganization in Subjects 1 and 4. The white arrow indicates additional pathways predicted in subject 5.

The CSTs of each subject, generated using deterministic tractography on the fODFs from CSD, are shown in Figure 3. In general, the CSTs at baseline and follow-up are similar, but interesting differences can be seen. The CSTs of subjects 1, 4, and 5 appear to cover a larger brain region at follow-up when compared to baseline (see arrows in figure). In subject 5, an alternate pathway (white arrow) from the internal capsule to the primary motor cortex is identified posteriorly. The CST of subject 4 appears to have a reorganized structure, particularly near the cortex (yellow arrow) when compared to the baseline tractogram. The CSTs of subject 2 are very similar at baseline and follow-up, with very little difference in breadth or shape. This subject also had the smallest degree of clinical change. In subject 3, there is a slight increase in the breadth of the CST on the affected side on follow-up.

**Figure 4:**
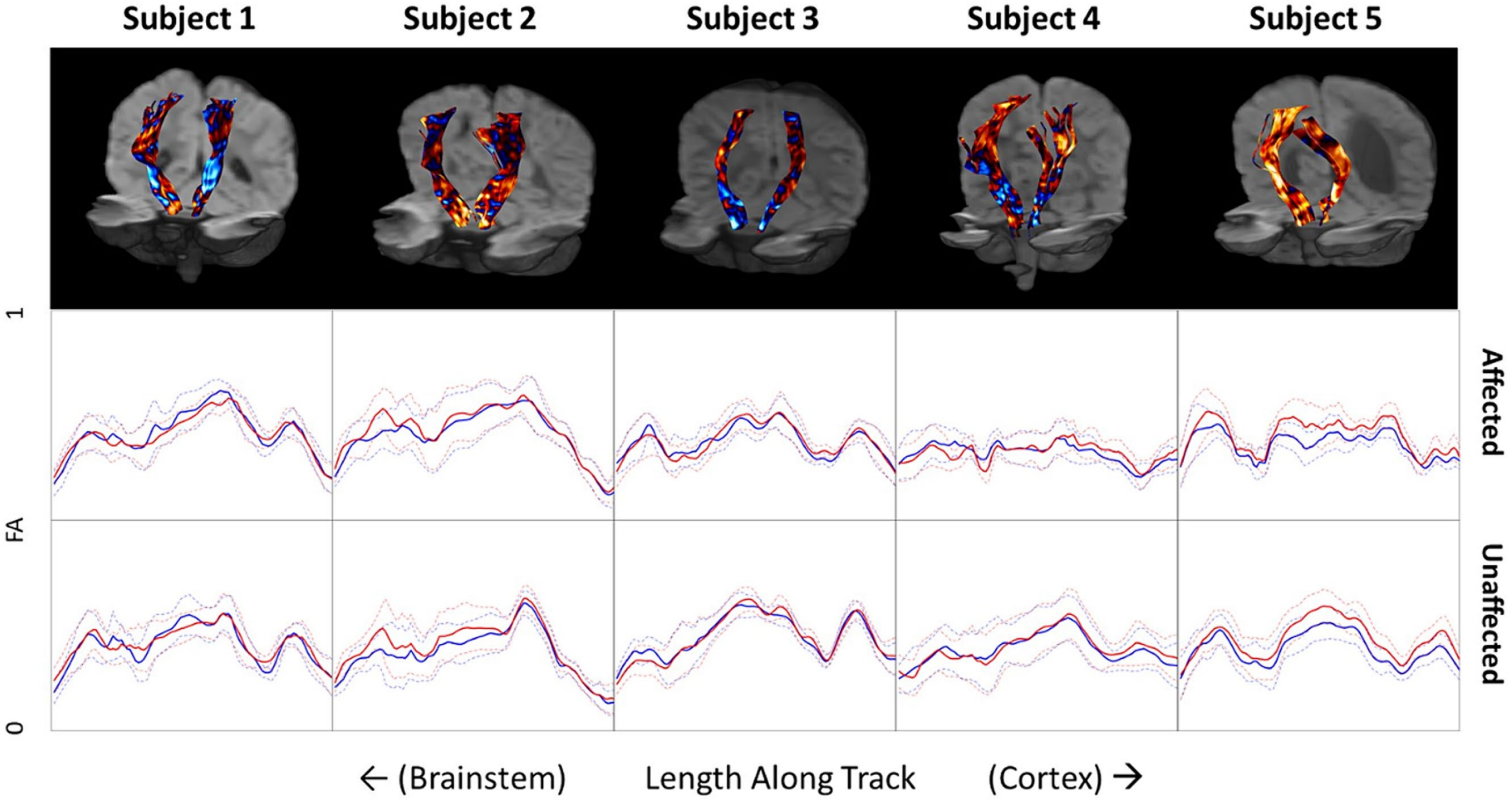
Top row: Differences in fractional anisotropy (FA) before and after therapy projected onto corticospinal tracts for all five subjects. Blue indicates a decrease in FA on follow-up. Red indicates an increase in FA on follow-up. Middle and bottom rows: FA values along the CST of the affected and unaffected side of the brain. Blue lines indicate baseline measurements before therapy and red lines indicate FA measures after therapy on follow-up. Dotted lines indicate the standard deviation of FA at each location.

The FA along all the streamlines in the CSTs were calculated and the differences between baseline and follow-up were measured in a common space and are color-encoded on the CSTs in the top row of Figure 4. Increases in FA are depicted in red and decreases in FA are depicted in blue. Beneath the tractograms, the raw values of FA at baseline and follow-up are plotted along the length of the CSTs on the affected and unaffected side. Each subject has unique pattern of FA along the CST, which are generally maintained after therapy. Subject 1 shows very little difference in FA before and after therapy along the entire length of the CST. Subjects 2 and 5 show slight but consistent increases in FA along the affected and unaffected CSTs. Subjects 3 and 4 show slight decreases in FA between the levels of the cerebral peduncles and the internal capsule and increases in FA as the CSTs course between the internal capsule and the primary motor cortex.

## Discussion

Given the young age and the mild to moderate severity of CP the five children, some improvement of motor function was expected. The goal of this small study was not to evaluate the therapy, but to investigate whether changes in motor function would correlate with imaging measures. Because of the improvements in clinically measured motor function in the five children studied, it could be expected to find changes in MRI-measured macrostructure and/or microstructure of the brain, specifically in the CSTs. Conventional MRI, i.e., T1-weighted and T2-FLAIR imaging, showed no identifiable changes after 36 weeks of therapy, as judged by two pediatric neuroradiologists (JM and UU). Tractography of the CST fiber bundles before and after 36 weeks of therapy show interesting differences, including increases in the spatial distribution of CST fiber bundles predicted by CSD tractography (See Figure 3). This is most prominent on the affected side but is also seen on the contralateral (unaffected) side. While this may be consistent with the idea of spreading neuronal connectivity with improvements in clinical scores, this interpretation should be made with a note of caution. Although the data obtained before and after therapy were processed identically, the results shown in Figure 3 are qualitative. The tractograms predicted by CSD tractography are a mathematical interpretation of the DMRI data through the fODFs produced by CSD analysis. Changes in the tractograms indicate differences in the diffusion properties, but do not necessarily correspond to alterations, appearance, or disappearance of physical neurite bundles.

FA values along the CST tractograms were calculated to try and obtain a more quantitative assessment of changes in microstructure along the CSTs. What is most striking about this data is how consistent it is across time. For instance, while each child shows a distinct pattern of FA along their CSTs, which is different on the affected and unaffected sides, they are strikingly consistent between baseline and follow-up. This consistency indicates a high degree of reliability of the imaging and processing methods but does not indicate that FA is a highly sensitive biomarker relating to clinical changes in motor function. It could be expected to see an increase in FA along the CSTs, the white matter bundles responsible for sending primary motor commands from the cortex to the musculoskeletal system, with improvement of motor function. Subject 5, who presented with a very large anatomical defect and associated porencephalic dilation of the left lateral ventricle at baseline and follow-up (Figures 1, 2), showed a 12.5-point increase in GMFM-66 and a 53% improvement in the Mobility subset of the PEDI-Functional Skills. Subject 5 also showed small but consistent increases in FA along the CST in both affected and unaffected sides. However, Subject 5 was also the youngest child studied and their young age might play a role in the changes observed in FA from baseline to follow-up. Additionally, other subjects showed a combination of both increases and decreases in FA after therapy and depended on the location along the CST.

While there are few studies that have reported on changes in FA and other white matter imaging metrics in this age group (29, 30) it is expected that as white matter matures in infancy and early childhood, FA would naturally increase. It is interesting, therefore, that decreases in FA are observed in some regions of the CSTs, despite the measured increase in motor function. Considerably more subjects are needed to assess the utility of serial DMRI for measuring macro-and microstructural progression and correlating the changes with clinical changes.

While there are several studies that have utilized DMRI to compare imaging findings of CP with those of healthy controls (29–32), there are very few that have looked at changes in imaging findings following intervention. Further, most studies using DMRI to evaluate CP were carried out in older children, not in toddler-aged children, as in this study.

Brain injury and structure in children with CP is highly variable and thus methods for assessment of images need to account for these individual differences. Even in this small set of children with hemiplegic CP, there is a high degree of variability in terms of brain injury and anatomy. When subjects within a study have similar gross neuroanatomy, each subject’s images can be registered to a common template and microstructural parameters from DMRI can be compared voxel-by-voxel (33). Even small differences between different groups can lead to significant statistical differences between groups. However, in the case of CP, the brains of each subject can be remarkably different in structure (Figure 1). Therefore, it is difficult to compare structures across multiple subjects as has been done previously (31, 32). In comparing the image features and CSTs in the five subjects in this small cohort (Figures 1, 3), the anatomy, the derived CSTs, and along tract FA profiles of each subject are substantially different. When this is the case, the metrics derived from DMRI may have limited utility for group analysis or for assessing the results across many subjects.

## Conclusion

In this brief research report, we have demonstrated that DMRI can be reliably carried out in toddler-aged children with mild-to-moderate spastic CP and that diffusion measures, including FA and tractograms can be generated. CST tractograms, measured before and after intensive physical and occupational therapy showed some differences in breadth and/or spatial footprint. However, these differences were not reflected in changes of FA along the CST, nor were they correlated with the magnitude of clinical improvements. While DMRI may be reliably carried out in children with CP, methods for quantitative comparison of results between children, who may have very different brain injury and anatomy, need further development.

## Data Availability

The raw data supporting the conclusions of this article will be made available by the authors, without undue reservation.
