# Supplementary material for: Changes in diffusion MRI and clinical motor function after physical/occupational therapies in toddler-aged children with spastic unilateral cerebral palsy
Source: Front Neurol. 2024 Oct 9;15:1418054. doi: 10.3389/fneur.2024.1418054 (PMC11496308; doi:10.3389/fneur.2024.1418054)
Supplement: Supplementary file 1 [file Data_Sheet_1.docx]

Supplementary Material

| **Sub#** | **Sex** | **Type of CNS Insult** | **Time of CNS Insult Indicated** | Clinic P-AA  (hours) | Outside Clinic  P-AA  (hours) |
| --- | --- | --- | --- | --- | --- |
| 1 | M | Stroke | Within the first year of life | 66.5 | 0 |
| 2 | M | Unknown | Within the first year of life | 71 | 16 |
| 3 | F | Stroke | During gestational or prenatal | 31 | 48 |
| 4 | F | Unknown | At the time of birth | 81 | 184 |
| 5 | M | Stroke | During gestational or prenatal | 35.5 | 350 |

**Table S1.** Subject demographics, insult information, and the amount of Perception–Action Approach (P-AA) physical and occupational therapy delivered in the clinic and at home.

**Table S2**. Child age, GMFCS, and GMFM-66 and PEDI Mobility scores at baseline and 36 wks. All subjects showed improvements in both tests and therefore the changes listed are always positive.

| **Sub#** | **Age at Consent (months)** | **GMFCS** | **GMFM-66** | | | **PEDI Mobility** | | | |
| --- | --- | --- | --- | --- | --- | --- | --- | --- | --- |
|  |  |  | **Baseline** | 36 wks. | Change | Baseline | 36 wks. | Change | %  change |
| 1 | 14 | 1 | 46.9 | 56.4 | 9.5 | 43.3 | 63.9 | 20.6 | 48% |
| 2 | 36 | 1 | 54.4 | 56.4 | 2 | 65 | 66.2 | 1.2 | 2% |
| 3 | 33 | 1 | 62.1 | 75.3 | 13.2 | 61.9 | 71.6 | 9.7 | 16% |
| 4 | 30 | 3 | 45.1 | 52.6 | 7.5 | 47.5 | 56.5 | 9 | 19% |
| 5 | 12 | 2 | 46.9 | 59.4 | 12.5 | 39.3 | 60 | 20.7 | 53% |
